# MARTINI-Based Protein-DNA Coarse-Grained HADDOCKing

**DOI:** 10.3389/fmolb.2019.00102

**Published:** 2019-10-01

**Authors:** Rodrigo V. Honorato, Jorge Roel-Touris, Alexandre M. J. J. Bonvin

**Affiliations:** ^1^Faculty of Science–Chemistry, Bijvoet Center for Biomolecular Research, Utrecht University, Utrecht, Netherlands; ^2^Brazilian Biosciences National Laboratory (LNBio), Brazilian Center for Research in Energy and Materials (CNPEM), Campinas, Brazil

**Keywords:** docking, biomolecular complexes, nucleic acids, coarse-graining, force field

## Abstract

Modeling biomolecular assemblies is an important field in computational structural biology. The inherent complexity of their energy landscape and the computational cost associated with modeling large and complex assemblies are major drawbacks for integrative modeling approaches. The so-called coarse-graining approaches, which reduce the degrees of freedom of the system by grouping several atoms into larger “pseudo-atoms,” have been shown to alleviate some of those limitations, facilitating the identification of the global energy minima assumed to correspond to the native state of the complex, while making the calculations more efficient. Here, we describe and assess the implementation of the MARTINI force field for DNA into HADDOCK, our integrative modeling platform. We combine it with our previous implementation for protein-protein coarse-grained docking, enabling coarse-grained modeling of protein-nucleic acid complexes. The system is modeled using MARTINI topologies and interaction parameters during the rigid body docking and semi-flexible refinement stages of HADDOCK, and the resulting models are then converted back to atomistic resolution by an atom-to-bead distance restraints-guided protocol. We first demonstrate the performance of this protocol using 44 complexes from the protein-DNA docking benchmark, which shows an overall ~6-fold speed increase and maintains similar accuracy as compared to standard atomistic calculations. As a proof of concept, we then model the interaction between the PRC1 and the nucleosome (a former CAPRI target in round 31), using the same information available at the time the target was offered, and compare all-atom and coarse-grained models.

## Introduction

Protein-DNA interactions play essential roles in cellular processes such as gene expression, regulation, transcription, DNA repair, or chromatin packaging in eukaryotes (Pandey et al., 2019). Computational docking, commonly referred to as prediction of the three-dimensional (3D) structure of a complex given the structures of its free constituents, has been extensively proven as an ideal complement to experimental structural methods in order to accurately model biomolecular complexes (Rodrigues and Bonvin, 2014). Even though computational modeling approaches have steadily progressed in the past decade (Janin, 2010), modeling large biomolecular assemblies still remains a challenge. In other words, application to either large individual or high number of interactors are limited by the significant computational cost of thoroughly sampling the complex and intricated conformational landscapes and by the increased difficulty of identifying near-native structures from the large pool of generated models (Rout and Sali, 2019).

Coarse-graining (CG) has been demonstrated to be a valuable alternative to standard atomistic (AA) approaches to alleviate some of those limitations and help the identification of the energy global minima by smoothing out the energy landscape (Hills et al., 2010; Roel-Touris et al., 2019). To this end, CG approaches group several atoms (either a few atoms or entire side chains) into larger “pseudo-atoms” or “beads,” which results into a reduction in the number of degrees of freedom of the system (Kmiecik et al., 2016). Historically, the development of CG force fields has followed two directions: (1) Physics-based, parametrized against its atomic counterpart or (2) knowledge-based, taking advantage of the increasing growth of statistical information derived from experimentally determined structures (Hills et al., 2010). Protein or/and protein-nucleic acid coarse-grained approaches have been implemented in several docking/modeling software such as for example: CABS-dock (Blaszczyk et al., 2016) RosettaDock (Gray et al., 2003), IMP (Russel et al., 2012), ATTRACT (Setny et al., 2012), NPDock (Tuszynska et al., 2015), PyRy3D (genesilico.pl/pyry3d), and more recently in HADDOCK (Dominguez et al., 2003; Roel-Touris et al., 2019), our integrative modeling platform.

MARTINI, a popular coarse-grained model for biomolecules, features lipids (Marrink et al., 2007) proteins (Monticelli et al., 2008), carbohydrates (López et al., 2009), and nucleic acids (Uusitalo et al., 2015, 2017) among others. Its DNA parametrization combines top-down (experimental data) and bottom-up (atomistic simulations) methodologies and is fully compatible with all other MARTINI models. On average, the nucleic acids' mapping follows a 1:6~7 rule, which means that each nucleotide is mapped onto six or seven CG beads. Bead types are selected according to partition free energies from water to chloroform or hydrated octanol. Bonded interactions have been fitted to reproduce dihedral, angle and bond distributions from atomistic simulations of short single stranded DNAs (ssDNAs) (Uusitalo et al., 2015). The general design and parametrization of MARTINI allow to easily combine several types of biomolecules (high transferability) as well as a straightforward conversion to atomistic resolution.

In this manuscript, we describe and benchmark the integration of the MARTINI coarse-grained force field for DNA into HADDOCK. It builds upon our recent implementation of a MARTINI coarse-grained protein-protein docking protocol (Roel-Touris et al., 2019) and is further optimized to account for Watson-Crick interactions. Prior to the docking, the input structures are converted into their coarse-grained counterparts and hydrogen-bonding base pairs are automatically detected so that a special set of parameters and restraints are used for those during the docking. We evaluate the performance of coarse-grained protein-nucleic acid docking using 44 unbound-unbound complexes from the protein-DNA benchmark (van Dijk and Bonvin, 2010). The results show a similar performance in terms of success rate and model quality while reducing the computational costs by ~6-fold compared to standard atomistic simulations. For 6 of those, we repeated the docking (both all-atom and coarse-grained) using experimental data to drive the docking as a demonstration that our coarse-grained protocol is also applicable for integrative modeling purposes. Finally, we showcase the potential of CG protein-DNA docking by revisiting the PRC1-nucleosome core particle complex (McGinty et al., 2014), which was offered as a CAPRI target (Target 95 in round 31; Lensink et al., 2017) for which we failed at the time to select any near native models.

## Methods

### Integration of the MARTINI DNA Coarse-Grained Force Field Into HADDOCK

The integration of the MARTINI coarse-grained force field for nucleic acids into HADDOCK builds upon our recent HADDOCK-CG implementation for protein-protein docking (Roel-Touris et al., 2019). We converted the MARTINI topologies and interaction parameters into a format compatible with the computational engine of HADDOCK, CNS–Crystallography and NMR System (Brünger et al., 1998). As in MARTINI, we represent the backbone of the nucleotide by three beads, one for the phosphate group, and two different beads for the sugar. Pyrimidines and purines are mapped into three and four beads, respectively. A detailed list of the topologies and parameters as used in HADDOCK can be found in the Supplementary Information (Tables SI-1, SI-2).

The latest official release of the MARTINI force field for nucleic acids, 2.2 (Uusitalo et al., 2015), includes eight additional beads and corresponding parameters compared to previous versions. These beads specifically account for Watson-Crick base pairing and mimics, to some extent, the hydrogen bonds that are formed between complementary nucleotide base pairs. These contribute to stabilizing the DNA double helix structure. When converting atomic structures into coarse-grained models, we automatically detect base pairing by calculating the Euclidean distance between neighboring nucleic acid side-chain atoms. We also use the distance between phosphate groups to ensure that bases are paired with their counterpart on the opposite strand and not with their neighbor in the sequence. We define a base pair when two opposite bases' heavy atoms are within the well-accepted hydrogen bond length of 3.5 Å, as used for example in LIGPLOT (Wallace et al., 1995), and their phosphate groups are at least 10 Å or further away from each other. If the input structures do not contain any phosphate, we use instead the center of mass of the nucleotides. By doing so, we avoid defining coupling between neighboring bases in sequence. This information is used by the HADDOCK machinery to ensure that specific interacting beads are used when necessary and the default HADDOCK DNA restraints were adapted to account for the CG beads and used to enforce correct DNA pairing (please see Table SI-3). As recommended in MARTINI, non-bonded interactions between CG beads are calculated using a 14 Å cutoff, whilst 8.5 Å is the default value for the united-atom OPLS force field (Jorgensen and Tirado-Rives, 1988) used in HADDOCK. Note that 8.5 Å is a reduced cutoff compared to the recommended one for OPLS, which was chosen as a compromise between accuracy and speed.

### Docking Procedure

Prior to the docking, we convert the atomic PDB coordinate files containing DNA/protein into a coarse-grained representation via an updated version of our in-house HADDOCK script for pre-processing CG input structures. During the vacuum part of the docking protocol (*it0* and *it1*) we set the dielectric constant (epsilon) to 78.0 to screen the high DNA charge (in the all atom representation). Epsilon is set to 1.0 for the final refinement stage in explicit solvent (*water*) (van Dijk and Bonvin, 2010). In the CG runs, the final water refinement is replaced by the back-mapping from coarse-grained to atomistic resolution as described in Roel-Touris et al. (2019). Note that in our atomistic DNA force field implementation the charge on the backbone phosphate is reduced to 0.5 since no counter ions are included in the docking to screen its charge, while the phosphate bead in MARTIN is uncharged. The final resulting models are clustered based on the fraction of common contacts (FCC) (Rodrigues et al., 2012) using a 0.6 cutoff (i.e., two models belonging to the same cluster share at least 60% of contacts) and a minimum of four models per cluster, which is the default clustering protocol in HADDOCK. All docking calculations were made using the latest 2.4 version of HADDOCK (still in beta version and unpublished but available upon request).

### Protein-DNA Docking Benchmark

To systematically test the performance of our coarse-grained implementation for protein-DNA docking, we used 44 unbound-unbound cases from the protein-DNA benchmark (van Dijk and Bonvin, 2008). Those are composed of 26 binary, 16 ternary, 1 quaternary (*2c5r*), and 1 pentameric (*1ddn*) complexes covering all major types of interactions (Luscombe et al., 2000). We removed three cases from the original dataset (PDB codes: *1diz, 1emh*, and *4ktq*) due to the fact that the MARTINI force field does not explicitly account for the modified nucleic bases P2U, NRI, and DOC. The benchmark is classified according to the amount of conformational changes that take place upon binding as measured by the interface positional root mean square deviation (i-RMSD) (i.e., unbound vs. bound structures) as follows:

- Easy (0 Å < i-RMSD ≤ 2 Å),
- Intermediate (2 Å < i-RMSD ≤ 5 Å), and
- Difficult (i-RMSD ≥ 5 Å).

This selection yielded 11 easy, 21 intermediate, and 12 difficult cases. For comparison purposes, we performed two different docking runs, one using the default atomistic force fields used by HADDOCK, and a second one with the parameters adapted from the MARTINI CG force field for both protein and DNA (Monticelli et al., 2008; Uusitalo et al., 2015). For the all-atom representation, OPLSX non-bonded parameters are used both for the protein (Jorgensen and Tirado-Rives, 1988) and DNA (Nozinovic et al., 2010). We used true interface information derived from the crystal structures translated into ambiguous interaction restraints (AIRs) to drive the docking calculations as previously defined in van Dijk and Bonvin (2010). The sampling parameters were kept to their default in HADDOCK: 1,000/200/200 models were generated for the rigid body (*it0*), simulated annealing (*it1*) and water refinement (*itw*) stages, respectively.

### Unbound Docking Using Experimental Data

We additionally modeled six complexes from the protein-DNA benchmark for which experimental data are available. The selected cases cover the different categories from the benchmark; “easy” (*1by4, 3cro*), “intermediate” (*1azp, 1jj4*), and “difficult” (*1a74, 1zme*). The available experimental information was collected from literature and include conserved residues, mutagenesis data, ethylation interference data, methylation interference data, NMR native state amide hydrogen exchange, and Raman spectroscopy as described in van Dijk and Bonvin (2010). As in the previous study (van Dijk and Bonvin, 2010), the sampling was slightly increased to 2,000/400/400 for *it0/it1/itw* docking stages, respectively.

### Modeling of the PRC1 Ubiquitylation Module Bound to the Nucleosome

We modeled the interaction between the multimeric PRC1 ubiquitylation module and the nucleosome by performing both AA and CG docking runs. As starting point for the docking, we used the unbound crystal structure of the enzymatical complex (PDB code: *3rpg*) and the nucleosome particle (PDB code: *3lz0*). We followed the same docking procedure as explained above (see Methods: Docking Procedure) except for the sampling parameters that were increased to 100,000, 400, and 400 for *it0, it1*, and *water* stages, respectively, because of the scarcity of the available information. The docking was driven by interaction restraints obtained from the literature at the time of CAPRI Round 31: One unambiguous distance restraint between the SG atom of the catalytic cysteine 85 of PRC1 and the NZ atoms of Lys119 or Lys118 on H2A, the ubiquitination target. In addition, we included mutagenesis data on PRC1 (K62A, R64A, K97A, and R98A) shown to be crucial for the interaction with the nucleosome (Bentley et al., 2011; Mattiroli et al., 2014). Ambiguous interaction restraints (AIRs) were defined for those (active) against all solvent accessible residues (passive) on the histones (those with either main chain or side chain relative accessibility >25% as calculated by NACCESS Lee and Richards, 1971). The list of active and passive residues used to guide the docking and the specific distance restraint can be found in Supplementary Information (Table SI-3).

### Metrics for the Evaluation of Model Quality

We evaluated the quality of the generated models following the standard CAPRI criteria (Janin, 2005). This includes the fraction of common contacts (Fnat) and the interface (i-RMSD) and ligand (l-RMSD) positional root mean square deviations from the reference crystal structures. Fnat is calculated from all heavy atom–heavy atom intermolecular contacts using a 5 Å distance cutoff. The i-RMSD is calculated on the interface backbone atoms after superimposition on the backbone of the interface residues, defined as those with any heavy atom within 10 Å distance of the partner molecule. The l-RMSD is calculated on the ligand backbone (usually the smallest molecule) after superimposition on the backbone atoms of the receptor (largest molecule). For both i-RMSD and l-RMSD, we only considered either backbone heavy atoms for atomistic models (C-alpha, C, N, O/P, C1, C9 for protein/DNA) or backbone particles (BB^*^) for coarse-grained models (in the *it0* and *it1* docking stages). The calculations were performed using ProFit (McLachlan, 1982) and the quality of the docking poses was classified as:

- High: Fnat ≥ 0.5 and (i-RMSD ≤ 1 Å or l-RMSD ≤ 1 Å),
- Medium: Fnat ≥ 0.3 and (1 Å < i-RMSD ≤ 2 or 1 Å < l-RMSD ≤ 5 Å),
- Acceptable: Fnat ≥ 0.1 Å and (2 Å < i-RMSD ≤ 4 Å or 5 Å < l-RMSD ≤ 10 Å), and
- Low: Fnat < 0.1 Å or (i-RMSD > 6 Å or l-RMSD > 10 Å).

### Metrics for the Evaluation of Docking Success Rate

We analyzed the performance of the docking calculations as: (1) The percentage of cases in which at least one model of a given accuracy is found within the top *N* solutions ranked by HADDOCK (*N* = 1, 5, 10, 20, 25, 50, 100, 200), and (2) the percentage of cases in which at least one acceptable or higher quality model was found in the top *T* clusters (*T* = 1, 2, 3, 4, 5).

## Results and Discussion

We have integrated the MARTINI CG force field for nucleic acids into HADDOCK version 2.4 (see Methods), combining it with our previous implementation of the protein MARTINI CG force field (Monticelli et al., 2008), enabling full coarse-grained protein-DNA docking. The AA to CG conversion scripts have been adapted to automatically account for specific Watson-Crick base pairing, which require special interacting parameters. In the following sections, we discuss the performance of our protocol for protein-DNA docking in terms of success rate and computational efficiency using 44 unbound-unbound complexes from the protein-DNA benchmark (van Dijk and Bonvin, 2008) with ideal interface information (see Methods; *Protein-DNA docking benchmark*). For six of them, we repeated the docking using experimental information to guide the docking. Finally, as a proof of concept, we revisited CAPRI Target 95 (Lensink et al., 2017), a protein-nucleosome complex for which we failed to identify near native solutions in our original CAPRI submissions (although we did generate some). In this new modeling, our top ranked predictions are in excellent agreement with the crystal structure of the complex (not used for the docking) for both standard atomistic docking and the hereby described coarse-grained implementation.

### Overall Performance of Coarse-Grained Protein-DNA Docking

The docking was performed starting from the unbound structures of each molecule and driven by AIRs as defined in our previous study (van Dijk and Bonvin, 2010; see Methods; Docking Procedure). In order to evaluate the performance of our approach, we calculated the success rates of both sets of runs (AA and CG) as the percentage of cases for which an acceptable or better quality was obtained in the top *N* ranked models (for details see Methods; Metrics for the Evaluation of Model Quality and Metrics for the Evaluation of Docking Success Rate).

**Figure 1:**
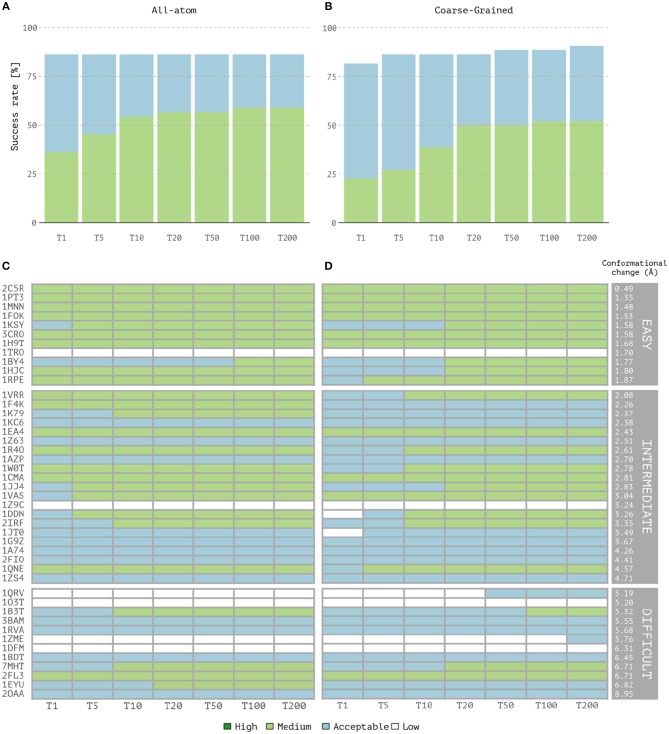
Performance of the all-atom and coarse-grained protocols in HADDOCK on the 44 unbound protein-DNA complexes of the benchmark. **(A)** Overall success rates (%) of the all-atom protocol on ranking single models as a function of the number of models considered. **(B)** Same as **(A)** but for the coarse-grained protocol. **(C,D)** The quality of the docking models for all 44 cases as a function of the number of models considered. The complexes are ordered by increasing degree of difficulty (from top to bottom) for both all-atom and CG docking runs. The color coding indicates the quality of the docked models according to CAPRI criteria.

Overall, coarse-grained docking generates and delivers acceptable or higher quality models for 40 out of the 44 cases after the back-mapping stage compared to 38 cases for the atomistic docking results. No near-native models are generated for four complexes; two of which are classified as difficult (*1dfm, 1o3t*), one as intermediate (*1z9c*) and one as easy (*1tro*). Inspection of the failed easy case reveals that it is a ternary complex (homodimer) and since no symmetry restraints were used in this case, its interface ambiguity was too high. In a previous benchmarking (van Dijk, 2006), acceptable models for this complex were obtained using a two-stage docking protocol in which a library of bent DNA conformations were given as input for the second docking run (a procedure not followed here). Among the successful CG cases, medium quality models are generated for 23 cases against 26 for the AA docking runs. Top one single structure-based ranking (best ranked structure) reaches 86.3% success rate for all-atom calculations vs. 81.8% for CG docking (Figures 1A,B). The overall success rates are similar for the top 5 and becomes higher for CG docking, reaching 90.9% in the top 200 while AA docking remains at 86.3% (which corresponds to 40 vs. 38 successful cases for CG and AA docking, respectively). In contrast, the quality of the models is slightly better for AA docking as measured by the success rates (Figures 1A,B) and rankings of medium quality models (Figures 1C,D). Notably, CG docking manages to generate acceptable models for two of the difficult cases that fail at standard atomistic HADDOCK runs (*1zme* and *1qrv*). In *1zme*, we find an acceptable model at position 176 (i.e., Top 200 according to our analysis) with 0.11/7.85 Å/9.94 Å for Fnat/i-RMSD/l-RMSD while the best AA model falls out the acceptable CAPRI criteria (0.04/7.51 Å/10.3 Å). For *1qrv*, the fourth case with the largest conformational change, the docked models generated by the standard AA HADDOCK protocol failed to satisfy the quality metric thresholds (Fnat and i-RMSD or Fnat and l-RMSD). However, several models showed a satisfactory overlap in terms of Fnat with >20% of interface contacts. With coarse-graining instead, the first acceptable model is found at rank 44 with a l-RMSD of 8.8 Å and Fnat of 0.14 (i.e., Top 50 according to our analysis).

Coarse-graining approaches benefit from the reduction of the number of degrees of freedom of the systems under study and make the docking calculations computationally more efficient. The median computational time to generate one model via CG in HADDOCK is 8.6s and of 42.8s for *it0* and *it1* stages, respectively, vs. 16.5s and 115.0s for standard atomistic calculations. Overall, the use of the MARTINI force field for both proteins and nucleic acids leads to a ~6-fold speed increase during rigid-body docking and semi-flexible stage (see SI-3, Table SI-5).

### Unbound Docking Using Experimental Data

We evaluated the capabilities of our HADDOCK-CG implementation to model protein-DNA interactions when using real experimental information. We selected six representative cases (van Dijk and Bonvin, 2010) from the protein-DNA benchmark classified as “easy” (*1by4, 3cro*), “intermediate” (*1azp, 1jj4*), and “difficult” (*1a74, 1zme*) for which experimental information was available. The latter was translated into AIRs (see Methods; Unbound Docking Using Experimental Data) in the form of *active* and *passive* residues and two different set of docking runs were performed using either the standard all-atom or the coarse-grained protocols.

As shown in Table 1, summarizing the quality of the generated clusters, for four out of the six cases, AA docking generates better quality models. No good solution in any of the tested protocols was found for *1zme*, which undergoes a large conformational change of 4.68 Å upon binding. In terms of sampling, the standard all-atom protocol, in combination with experimental data, generates ~900 near-native models (i.e., acceptable or higher quality according to CAPRI) on average per case, while our CG approach around three times less (~300). This is somewhat surprising as the smoother energy landscape derived from the reduction of degrees of freedom might help the sampling process as previously demonstrated in our protein-protein CG implementation (Roel-Touris et al., 2019). Despite this difference in sampling, both approaches perform rather similarly in terms of structure quality, indicating that our CG protocol is also applicable for integrative modeling of complexes in combination with real experimental data. Recent studies have indicated that the interpretation of CG models using experimental data, and in particular SAXS data, can benefit from improved forward models as demonstrated by Paissoni et al. (2019) for protein-DNA complexes.

**Table 1:** Performance of the all-atom and coarse-grained protocols in HADDOCK on six representative cases of the protein-DNA benchmark using experimental data to drive the docking.

	**All-Atom**					**Coarse-Grained**				
**Complex**	**Cluster**	**i-RMSD**	**l-RMSD**	**Fnat**	**CAPRI**	**Cluster**	**i-RMSD**	**l-RMSD**	**Fnat**	**CAPRI**
**EASY**										
1BY4	2nd	3.66	14.37	0.18	*	1st	3.08	9.05	0.19	*
3CRO	1st	1.52	2.34	0.39	**	2nd	2.77	7.35	0.22	*
**INTERMEDIATE**										
1AZP	1st	3.14	10.16	0.11	*	1st	3.53	9.29	0.10	*
1JJ4	2nd	1.98	5.71	0.25	*	1st	2.24	6.55	0.11	*
**DIFFICULT**										
1A74	1st	1.61	4.41	0.32	**	1st	1.83	4.54	0.24	*
1ZME	1st	8.52	29.54	0.00	–	1st	8.4	30.7	0.00	–

The RMSDs (Å) and Fnats correspond to the best model of the best cluster. The ranking of the best cluster is also reported. The CAPRI column indicates the number of models per quality threshold (*acceptable, **medium, ***high).

### Revisiting CAPRI Target 95: The PRC1 Ubiquitination Module Bound to the Nucleosome

The polycomb repressive complex 1 (PRC1) represses the expression of genes regulated by developmental processes and is responsible for the ubiquitylation of the nucleosomal histone (Mattiroli et al., 2014). This complex was offered as a blind target to the CAPRI experiment (Round 31, target 95), to which we participated but failed to correctly identify near-native models out of our pool of generated complexes. Using the same information derived from the literature as used in CAPRI Round 31 (see Table SI-4), we repeated the docking using our MARTINI implementation in HADDOCK2.4 and validated our predictions against the crystal structure of the complex (PDB-ID: *4rp8*; McGinty et al., 2014).

**Figure 2:**
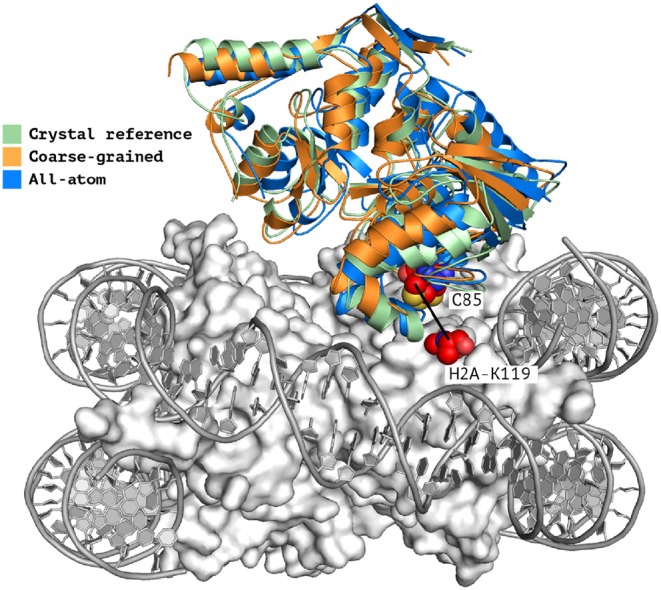
Single structure comparison of top-ranking models predicted by HADDOCK. Superimposition of the best models (top-ranked) predicted by HADDOCK using atomistic (blue) or coarse-grained (orange) docking onto the experimental crystal structure (PDB-ID 4r8p, green; McGinty et al., 2014). The two residues PRC1-Cys85 and H2A-Lys119 which are expected to form a covalent bond (Kerscher et al., 2006; an information used to guide the docking) are shown as spheres. The interface RMSD of the all-atom and coarse-grained top rankings models against the reference crystal structure are 3.23 and 3.0 Å, respectively.

When analyzing the i-RMSD of the top-ranked model according to the HADDOCK score, the CG one is slightly closer (3.0 Å) to the reference crystal structure than the corresponding AA model (3.14 Å; Table 2A). Same behavior is observed when looking at the clustering statistics, in which the average i-RMSD for the top four models of the best cluster for CG was 3.09 ± 0.08 Å against 3.23 ± 0.23 Å in AA. A much large difference between the two protocols is however clearly visible when comparing the number of acceptable of better models generated at the various docking stages (Table 2B) with CG docking resulting in ~1.5 times more acceptable models than AA docking. This improvement in the sampling is in contrast to what was observed above for the protein-DNA benchmark. As already observed for protein-protein docking (Roel-Touris et al., 2019), the impact of coarse graining is more evident when little or no information (*ab-initio* docking) is available to drive the docking process. Finally, a view of the top ranked models superimposed onto the reference crystal structure is shown in Figure 2. Both satisfy the distance restraint imposed to model the interaction between Cys85 of PRC1 with Lys118/119 of Histone 2A (PRC1-H2A). The proximity of those two residues was proposed (Bentley et al., 2011) to be necessary to restrict the ligase complex to a single region of the nucleosome (the information we used in CAPRI), which was confirmed by the crystal structure (PDB-ID 4r8p; McGinty et al., 2014).

**Table 2A:** Sampling and quality assessment of the AA and CG PRC1 docking models.

	**# of acceptable models**			**Time per model [s]**	
	**it0^a^**	**it1**	**Water**	**it0**	**it1**
All-atom	360/173	169	169	138	979
Coarse-grained	536/293	290	254	27	188

Number Of Acceptable Models And Time Necessary To Generate One Model For The Rigid-Body And Semi-Flexible Stages For Both All-Atom And Coarse-Grained Simulations.

a *The first number is the total number of acceptable models within the 10,000 generated and the second correspond to those in the top400 selected for further semi-flexible refinement*.

**Table 2B:** Ranking, i-RMSD Comparison And Time Per Model Of All-Atom And Coarse-Grained Simulation Of Capri Target 95.

	**Single structure**		**Cluster**	
	**Rank**	**i-RMSD [Å]**	**Rank**	**Top4 < i-RMSD> [Å]**
All-Atom	1	3.14	2	3.23 ± 0.23
Coarse-grained	1	3.00	1	3.09 ± 0.08

## Conclusion

In this work, we have presented the integration of the MARTINI coarse-grained force field for nucleic acids into our HADDOCK integrative modeling software. It builds upon our previous implementation for protein-protein docking, using a coarse-grained representation during the rigid-body and semi-flexible refinement stages, and converting back the resulting models to atomistic resolution following an atom-to-bead distance restrained-guided morphing procedure. We have shown that the performance of coarse-grained docking is similar to that of standard all-atom protocol in terms of success rate, while the quality of the generated models remains rather similar according to standard CAPRI criteria. We demonstrated that our coarse-grained protocol is perfectly suited for use with experimental or predicted data. In particular, we have revisited a challenging target of the CAPRI experiment, taking full advantage of the hereby described implementation and obtaining near-native models of PRC1 Ubiquitination module bound to the nucleosome in excellent agreement with the crystal reference. Further, by smoothening the energy landscape it also allows to generate more near native models in cases where limited information is available to guide the modeling, which should also benefit the scoring stage since it becomes easier to identify them. It also brings a significant gain in computing performance, with a ~6-fold speed increase compared to standard atomistic simulations. In conclusion, with this extension, HADDOCK has gained the capability to model significantly larger assemblies consisting of mixed protein and DNA components, in a more efficient way without compromising its overall performance.

## Data Availability Statement

The datasets generated for this study are available on request to the corresponding author.

## Author Contributions

AB, RH, and JR-T conceived and designed the research and wrote the paper. RH and JR-T performed the computational analysis and interpreted the data.

### Conflict of Interest

The authors declare that the research was conducted in the absence of any commercial or financial relationships that could be construed as a potential conflict of interest.

## References

[B1] Bentley M. L. Corn J. E. Dong K. C. Phung Q. Cheung T. K. Cochran A. G. (2011). Recognition of UbcH5c and the nucleosome by the Bmi1/Ring1b ubiquitin ligase complex: E2 and substrate recognition by Bmi1/Ring1b. EMBO J. 30, 3285–3297. 10.1038/emboj.2011.243 21772249 PMC3160663

[B2] Blaszczyk M. Kurcinski M. Kouza M. Wieteska L. Debinski A. Kolinski A.. (2016). Modeling of protein–peptide interactions using the CABS-dock web server for binding site search and flexible docking. Methods 93, 72–83. 10.1016/j.ymeth.2015.07.004 26165956

[B3] Brünger A. T. Adams P. D. Clore G. M. DeLano W. L. Gros P. Grosse-Kunstleve R. W.. (1998). Crystallography and NMR system: a new software suite for macromolecular structure determination. Acta Crystallogr. D Biol. Crystallogr. 54, 905–921. 10.1107/S0907444998003254 9757107

[B4] Dominguez C. Boelens R. Bonvin A. M. J. J. (2003). HADDOCK: a protein–protein docking approach based on biochemical or biophysical information. J. Am. Chem. Soc. 125, 1731–1737. 10.1021/ja026939x 12580598

[B5] Gray J. J. Moughon S. Wang C. Schueler-Furman O. Kuhlman B. Rohl C. A.. (2003). Protein–protein docking with simultaneous optimization of rigid-body displacement and side-chain conformations. J. Mol. Biol. 331, 281–299. 10.1016/S0022-2836(03)00670-3 12875852

[B6] Hills R. D. Lu L. Voth G. A. (2010). Multiscale coarse-graining of the protein energy landscape. PLoS Comput. Biol. 6:e1000827. 10.1371/journal.pcbi.1000827 20585614 PMC2891700

[B7] Janin J. (2005). Assessing predictions of protein-protein interaction: the CAPRI experiment. Protein Sci. 14, 278–283. 10.1110/ps.041081905 15659362 PMC2253420

[B8] Janin J. (2010). Protein–protein docking tested in blind predictions: the CAPRI experiment. Mol. BioSyst. 6:2351. 10.1039/c005060c 20725658

[B9] Jorgensen W. L. Tirado-Rives J. (1988). The OPLS [optimized potentials for liquid simulations] potential functions for proteins, energy minimizations for crystals of cyclic peptides and crambin. J. Am. Chem. Soc. 110, 1657–1666. 10.1021/ja00214a001 27557051

[B10] Kerscher O. Felberbaum R. Hochstrasser M. (2006). Modification of proteins by ubiquitin and ubiquitin-like proteins. Annu. Rev. Cell Dev. Biol. 22, 159–180. 10.1146/annurev.cellbio.22.010605.093503 16753028

[B11] Kmiecik S. Gront D. Kolinski M. Wieteska L. Dawid A. E. Kolinski A. (2016). Coarse-grained protein models and their applications. Chem. Rev. 116, 7898–7936. 10.1021/acs.chemrev.6b00163 27333362

[B12] Lee B. Richards F. M. (1971). The interpretation of protein structures: estimation of static accessibility. J. Mol. Biol. 55, 379–400. 10.1016/0022-2836(71)90324-X 5551392

[B13] Lensink M. F. Velankar S. Wodak S. J. (2017). Modeling protein-protein and protein-peptide complexes: CAPRI 6th edition: modeling protein-protein and protein-peptide complexes. Proteins 85, 359–377. 10.1002/prot.25215 27865038

[B14] López C. A. Rzepiela A. J. de Vries A. H. Dijkhuizen L. Hünenberger P. H. Marrink S. J. (2009). Martini coarse-grained force field: extension to carbohydrates. J. Chem. Theory Comput. 5, 3195–3210. 10.1021/ct900313w 26602504

[B15] Luscombe N. M. Austin S. E. Berman H. M. Thornton J. M. (2000). An overview of the structures of protein-DNA complexes. Genome Biol. 1:REVIEWS001. 10.1186/gb-2000-1-1-reviews001 11104519 PMC138832

[B16] Marrink S. J. Risselada H. J. Yefimov S. Tieleman D. P. de Vries A. H. (2007). The MARTINI force field: coarse grained model for biomolecular simulations. J. Phys. Chem. B 111, 7812–7824. 10.1021/jp071097f 17569554

[B17] Mattiroli F. Uckelmann M. Sahtoe D. D. van Dijk W. J. Sixma T. K. (2014). The nucleosome acidic patch plays a critical role in RNF168-dependent ubiquitination of histone H2A. Nat. Commun. 5:3291. 10.1038/ncomms4291 24518117 PMC3929782

[B18] McGinty R. K. Henrici R. C. Tan S. (2014). Crystal structure of the PRC1 ubiquitylation module bound to the nucleosome. Nature 514, 591–596. 10.1038/nature13890 25355358 PMC4215650

[B19] McLachlan A. D. (1982). Rapid comparison of protein structures. Acta Cryst. A 38, 871–873. 10.1107/S0567739482001806

[B20] Monticelli L. Kandasamy S. K. Periole X. Larson R. G. Tieleman D. P. Marrink S.-J. (2008). The MARTINI coarse-grained force field: extension to proteins. J. Chem. Theory Comput. 4, 819–834. 10.1021/ct700324x 26621095

[B21] Morin A. Eisenbraun B. Key J. Sanschagrin P. C. Timony M. A. Ottaviano M.. (2013). Collaboration gets the most out of software. eLife 2:e01456. 10.7554/eLife.01456 24040512 PMC3771563

[B22] Nozinovic S. Fürtig B. Jonker H. R. A. Richter C. Schwalbe H. (2010). High-resolution NMR structure of an RNA model system: the 14-mer cUUCGg tetraloop hairpin RNA. Nucleic Acids Res. 38, 683–694. 10.1093/nar/gkp956 19906714 PMC2811024

[B23] Paissoni C. Jussupow A. Camilloni C. (2019). Martini bead form factors for nucleic acids and their application in the refinement of protein–nucleic acid complexes against SAXS data. J. Appl. Crystallogr. 52, 394–402. 10.1107/S1600576719002450

[B24] Pandey P. Hasnain S. Ahmad S. (2019). Protein-DNA interactions, in Encyclopedia of Bioinformatics and Computational Biology, eds Ranganathan S. Gribskov M. Nakai K. Schönbach C. (Academic Press), 142–154. 10.1016/B978-0-12-809633-8.20217-3

[B25] Rodrigues J. P. G. L. M. Bonvin A. M. J. J. (2014). Integrative computational modeling of protein interactions. FEBS J. 281, 1988–2003. 10.1111/febs.12771 24588898

[B26] Rodrigues J. P. G. L. M. Trellet M. Schmitz C. Kastritis P. Karaca E. Melquiond A. S. J.. (2012). Clustering biomolecular complexes by residue contacts similarity. Proteins 80, 1810–1817. 10.1002/prot.24078 22489062

[B27] Roel-Touris J. Don C. G. Honorato R. V. Rodrigues J. P. G. L. M. Bonvin A. M. J. J. (2019). Less is more: Coarse-grained integrative modeling of large biomolecular assemblies with HADDOCK. J. Chem. Theory Comput. 10.1101/715268 [Epub ahead of print]. 31539250 PMC6854652

[B28] Rout M. P. Sali A. (2019). Principles for integrative structural biology studies. Cell 177, 1384–1403. 10.1016/j.cell.2019.05.016 31150619 PMC6810593

[B29] Russel D. Lasker K. Webb B. Velázquez-Muriel J. Tjioe E. Schneidman-Duhovny D.. (2012). Putting the pieces together: integrative modeling platform software for structure determination of macromolecular assemblies. PLoS Biol. 10:e1001244. 10.1371/journal.pbio.1001244 22272186 PMC3260315

[B30] Setny P. Bahadur R. P. Zacharias M. (2012). Protein-DNA docking with a coarse-grained force field. BMC Bioinformatics 13:228. 10.1186/1471-2105-13-228 22966980 PMC3522568

[B31] Tuszynska I. Magnus M. Jonak K. Dawson W. Bujnicki J. M. (2015). NPDock: a web server for protein–nucleic acid docking. Nucleic Acids Res. 43, W425–W430. 10.1093/nar/gkv493 25977296 PMC4489298

[B32] Uusitalo J. J. Ingólfsson H. I. Akhshi P. Tieleman D. P. Marrink S. J. (2015). Martini coarse-grained force field: extension to DNA. J. Chem. Theory Comput. 11, 3932–3945. 10.1021/acs.jctc.5b00286 26574472

[B33] Uusitalo J. J. Ingólfsson H. I. Marrink S. J. Faustino I. (2017). Martini coarse-grained force field: extension to RNA. Biophys. J. 113, 246–256. 10.1016/j.bpj.2017.05.043 28633759 PMC5529176

[B34] van Dijk M. (2006). Information-driven protein-DNA docking using HADDOCK: it is a matter of flexibility. Nucleic Acids Res. 34, 3317–3325. 10.1093/nar/gkl412 16820531 PMC1500871

[B35] van Dijk M. Bonvin A. M. J. J. (2008). A protein-DNA docking benchmark. Nucleic Acids Res. 36, e88. 10.1093/nar/gkn386 18583363 PMC2504314

[B36] van Dijk M. Bonvin A. M. J. J. (2010). Pushing the limits of what is achievable in protein–DNA docking: benchmarking HADDOCK's performance. Nucleic Acids Res. 38, 5634–5647. 10.1093/nar/gkq222 20466807 PMC2943626

[B37] Wallace A. C. Laskowski R. A. Thornton J. M. (1995). LIGPLOT: a program to generate schematic diagrams of protein-ligand interactions. Protein Eng. Des. Sel. 8, 127–134. 10.1093/protein/8.2.127 7630882

