# Supplementary material for: MARTINI-Based Protein-DNA Coarse-Grained HADDOCKing
Source: Front Mol Biosci. 2019 Oct 1;6:102. doi: 10.3389/fmolb.2019.00102 (PMC6779769; doi:10.3389/fmolb.2019.00102)
Supplement: Supplementary file 1 [file Data_Sheet_1.PDF]

Supplementary Information

Protein-DNA coarse-grained docking with HADDOCK

Rodrigo V. Honorato[1,2] ‡, *Jorge Roel-Touris*[1] ‡, *Alexandre M.J.J. Bonvin*[1*]

[1]Bijvoet Center for Biomolecular Research, Faculty of Science – Chemistry, Utrecht University. The Netherlands.

[2]Brazilian Biosciences National Laboratory (LNBio), Brazilian Center for Research in Energy and Materials (CNPEM), Zip Code 13083-970, Campinas, São Paulo, Brazil.

‡Joint first authors as both contributed equally.

*To whom correspondence should be addressed. Email: a.m.j.j.bonvin@uu.nl

TABLE OF CONTENTS

SI-1. Implementation of the MARTINI force field for DNA in HADDOCK …..…………… 3

Table SI-1. Nucleotide particle types.

Table SI-2. Nucleotide particle relations.

Table SI-3. Nucleotide particle relations for the special H-bonding beads.

SI-2. Modeling of the PRC1 ubiquitylation module bound to the nucleosome ……………… 5

Table SI-4. Ambiguous and unambiguous interaction restraints as defined in HADDOCK for the modeling of PRC1-nucleosome complex.

SI-3. Detailed overview of the selected cases from the protein-DNA benchmark ………….. 6

Table SI-5. Atom/Bead count and computing time for the selected cases of the protein-DNA benchmark.

SI-1. IMPLEMENTATION OF THE MARTINI FORCE FIELD FOR DNA IN HADDOCK

Converted force field parameters from the original work of *Uusitalo. et al* into CNS compatible format.

Table SI-1. Nucleotide particle types.

Nucleotide	Backbone	Side-chains	H-bonding
ADE	NB1-NB2-NB3	ANS1-ANS1-ANS3-ANS2	NH1-NH2
GUA	NB1-NB2-NB3	GNS1-GNS3-GNS4-GNS2	NH3-NH4
THY	NB1-NB2-NB3	TNS1-TNS4-TNS2	NH5-NH6
CYT	NB1-NB2-NB3	CNS1-CNS4-CNS3	NH7-NH8

Table SI-2. Nucleotide particle relations.

A) Adapted bond length parameters and corresponding force constants. D: Bond Length (Å). K: Force Constant (kcal.mol^{-1}).

Nucleotide	$D_{BB1-BB2}$ (K)	$D_{BB2-BB3}$ (K)	$D_{BB3-SC1}$ (K)	$D_{SC1-SC2}$ (K)	$D_{SC2-SC3}$ (K)	$D_{SC2-SC4}$ (K)	$D_{SC3-SC4}$ (K)	$D_{SC4-SC1}$ (K)
ADE	3.6 (47.87)	1.98 (191.479)	3.0 (71.805)	2.29 (500.0)	2.66 (500.0)	3.26 (47.87)	2.88 (500.0)	1.62 (500.0)
GUA	3.6 (47.87)	1.98 (191.479)	3.0 (71.805)	2.95 (500.0)	2.95 (500.0)	3.89 (47.87)	2.85 (500.0)	1.61 (500.0)
THY	3.6 (47.87)	1.98 (191.479)	2.7 (71.805)	2.17 (500.0)	3.22 (500.0)	–	–	2.65* (500.0)
CYT	3.6 (47.87)	1.98 (191.479)	2.7 (71.805)	2.2 (500.0)	2.85 (500.0)	–	–	2.68* (500.0)

*$D_{SC3-SC1}$

B) Adapted bond angle parameters and corresponding force constants. ϕ: Bond Angle (°). K: Force Constant (kcal.mol^{-1}).

Nucleotide	$\phi_{BB1-BB2-BB3}$ (K)	$\phi_{BB2-BB3-SC1}$ (K)	$\phi_{BB3-SC1-SC2}$ (K)	$\phi_{BB3-SC1-SC4}$ (K)	$\phi_{SC1-SC2-SC3}$ (K)	$\phi_{SC2-SC1-SC4}$ (K)	$\phi_{SC2-SC3-SC4}$ (K)	$\phi_{SC3-SC4-SC1}$ (K)
ADE	110 (47.87)	94 (59.84)	160 (47.87)	140 (47.87)	85 (47.87)	125 (47.87)	74 (47.87)	98 (47.87)
GUA	110 (47.87)	94 (59.84)	137 (71.8)	130 (59.84)	69 (47.87)	125 (47.87)	84 (47.87)	94 (47.87)
THY	110 (47.87)	92 (52.66)	107 (71.8)	145* (71.8)	55 (23.93)	83** (23.93)	42*** (23.93)	–
CYT	110 (47.87)	95 (50.26)	95 (71.8)	150* (71.8)	61 (47.87)	71** (47.87)	47*** (47.87)	–

*$\phi_{BB2-SC1-SC3}$, **$\phi_{SC2-SC1-SC3}$, ***$\phi_{SC2-SC3-SC1}$

C) Adapted bond dihedral parameters and corresponding force constants. Ψ: Bond Dihedral (°). K: Force Constant (kcal.mol^{-1}).

Nucleotide	$\Psi_{BB1-BB2-BB3-SC1}$ (K)	$\Psi_{BB2-BB3-SC1-SC2}$ (K)	$\Psi_{BB2-BB3-SC1-SC4}$ (K)
ADE	-90 (0.05)	-116 (0.0)	98 (0.04)
GUA	-90 (0.05)	-117 (0.0)	92 (0.04)
THY	-75 (0.1)	-110 (0.04)	-145* (0.16)
CYT	-78 (0.05)	-90 (0.05)	-142* (0.12)

*$\Psi_{BB2-BB3-SC1-SC3}$

Table SI-3. Nucleotide particle relations for the special H-bonding beads.

For the sake of simplicity, regardless the nucleotide the special beads are displayed as NH1 and NH2.

A) Adapted bond length parameters and corresponding force constants.
D: Bond Length (Å). K: Force Constant (kcal.mol^{-1}).

Nucleotide	$D_{SC2-NH1}$ (K)	$D_{SC3-NH1}$ (K)	$D_{SC4-NH1}$ (K)	$D_{SC2-NH2}$ (K)	$D_{SC4-NH2}$ (K)	$D_{NH1-NH2}$ (K)
**ADE**	2.29 (500.0)	2.66 (500.0)	3.26 (47.87)	2.66 (500.0)	2.88 (500.0)	2.66 (500.0)
**GUA**	2.95 (500.0)	2.95 (500.0)	3.89 (47.87)	2.95 (500.0)	2.85 (500.0)	2.95 (500.0)
**THY**	2.17 (500.0)	3.22 (500.0)	-	2.65 (500.0)	3.22 (500.0)	3.22 (500.0)
**CYT**	2.2 (500.0)	2.85 (500.0)	-	2.68 (500.0)	2.85 (500.0)	2.85 (500.0)

B) Adapted bond angle parameters and corresponding force constants. ϕ: Bond Angle (°).
K: Force Constant (kcal.mol^{-1}).

Nucleotide	$\phi_{BB3-SC1-NH1}$ (K)	$\phi_{SC2-SC3-NH1}$ (K)	$\phi_{SC2-SC4-NH1}$ (K)	$\phi_{SC2-NH1-NH2}$ (K)	$\phi_{SC3-SC4-NH1}$ (K)	$\phi_{SC4-NH1-NH2}$ (K)	$\phi_{SC1-SC2-NH2}$ (K)	$\phi_{SC2-SC4-NH2}$ (K)
**ADE**	160 (47.87)	85 (47.87)	125 (47.87)	85 (47.87)	74 (47.87)	74 (47.87)	85 (47.87)	74 (47.87)
**GUA**	137 (71.80)	84 (47.87)	125 (47.87)	69 (47.87)	69 (47.87)	84 (47.87)	94 (47.87)	94 (47.87)
**THY**	107 (71.80)	83 (23.93)	55* (23.93)	83 (23.93)	-	42** (23.93)	83 (23.93)	55*** (23.93)
**CYT**	95 (71.80)	71 (47.87)	47* (47.87)	71 (47.87)	-	47** (47.87)	71 (47.87)	71*** (47.87)

*$\phi_{SC2-SC3-NH1}$, **$\phi_{SC3-NH1-NH2}$, ***$\phi_{SC2-SC3-NH2}$

C) Adapted bond dihedral parameters and corresponding force constants.
Ψ: Bond Dihedral (°). K: Force Constant (kcal.mol^{-1}).

Nucleotide	$\Psi_{BB2-BB3-SC1-NH1}$ (K)	$\Psi_{BB2-BB3-SC1-NH2}$ (K)
**ADE**	-116 (0.0)	-
**GUA**	-117 (0.0)	-
**THY**	-110 (0.0)	-145 (0.16)
**CYT**	-90 (0.05)	-142 (0.12)

SI-2. MODELING OF THE PRC1 UBIQUITYLATION MODULE BOUND TO THE NUCLEOSOME

Table SI-4. Ambiguous and unambiguous interaction restraints as defined in HADDOCK for the modeling of PRC1-nucleosome complex.

Type		Residue
Specific distance restraint		H2A: Cys85 (Atom SG) - PRC1: Lys118, Lys119 (Atom NZ). Distance range 0-2Å
Ambiguous Interaction Restraints (AIRs)	Active	62, 64, 97, 98
	Passive	38, 40, 52, 53, 56, 59, 64, 76, 77, 78, 80, 81, 86, 105, 115, 125, 129, 132, 134, 220, 221, 223, 224, 227, 231, 249, 252, 259, 260, 264, 267, 277, 284, 293, 294, 419, 422, 435, 436, 441, 468, 471, 472, 473, 474, 489, 491, 495, 498, 499, 509, 510, 513, 514, 516, 517, 518, 519, 628, 629, 631, 640, 644, 647, 648, 653, 654, 682, 696, 701, 702, 705, 706, 709, 712, 713, 716, 717, 719, 720, 722, 839, 852, 853, 856, 859, 864, 869, 876, 877, 880, 881, 886, 890, 915, 922, 925, 929, 932, 933, 934, 935, 1025, 1027, 1031, 1048, 1049, 1052, 1056, 1059, 1060, 1074, 1077, 1084, 1091, 1093, 1101, 1214, 1215, 1219, 1222, 1236, 1241, 1268, 1271, 1272, 1273, 1274, 1289, 1291, 1295, 1298, 1299, 1309, 1310, 1313, 1314, 1316, 1317, 1318, 1319, 1320, 1429, 1431, 1432, 1440, 1444, 1447, 1448, 1453, 1454, 1468, 1476, 1482, 1489, 1496, 1501, 1502, 1505, 1506, 1509, 1510, 1512, 1513, 1516, 1517, 1519, 1520, 1521

SI-3. DETAILED OVERVIEW OF THE SELECTED CASES FROM THE PROTEIN-DNA DOCKING BENCHMARK

Table SI-5. Atom/Bead count and computing time for the rigid-body and semi-flexible refinement stages (*it0* + *it1*) for the selected cases of the protein-DNA benchmark. CPU times are averaged values (seconds/model)[#]

Case	Atom count	Bead count	<AA time>	<CG time>
**1AZP**	1175	300	32	25
**1PT3**	1845	513	55	27
**2IRF**	1925	493	78	21
**1QRV**	2107	554	64	31
**1HJC**	2158	547	62	23
**1VAS**	2275	581	61	28
**1K79**	2459	631	67	30
**1W0T**	2476	614	93	35
**1ZME**	2558	654	92	29
**1RPE**	2558	643	89	45
**1JJ4**	2603	697	78	31
**1R4O**	2686	652	132	33
**3CRO**	2696	664	92	47
**1QNE**	2703	705	247	32
**1CMA**	2710	740	71	31
**1BY4**	2713	699	94	40
**1EA4**	3003	770	99	40
**2FL3**	3084	862	198	41
**2OAA**	3169	897	90	36
**1TRO**	3273	824	130	45
**1BDT**	3648	923	140	53
**1B3T**	3658	959	111	56
**1MNN**	3793	1058	157	57
**1F4K**	3827	995	125	56
**7MHT**	3957	1102	369	50
**1EYU**	3969	1078	311	57
**1KSY**	4236	1140	130	55
**1A74**	4439	1180	138	73
**2C5R**	4459	1280	304	84
**1ZS4**	4576	1163	198	51
**1G9Z**	4593	1248	316	77
**1Z9C**	4613	1190	168	66
**1VRR**	4633	1260	202	72
**3BAM**	4733	1354	174	64
**2FIO**	5038	1265	175	56
**1DFM**	5460	1540	182	67
**1H9T**	5577	1519	194	96
**1KC6**	5638	1616	263	98
**1RVA**	5761	1672	193	104
**1O3T**	5930	1551	201	60
**1Z63**	6022	1664	158	69
**1FOK**	6854	1900	291	110
**1DDN**	7611	1994	710	179
**1JT0**	9476	2704	262	179

The timing corresponds to the total time in seconds reported by CNS as measured on an AMD Opteron (tm) Processor 6344.
